# Efficacy of an automated laser for reducing wild bird visits to the free range area of a poultry farm

**DOI:** 10.1038/s41598-021-92267-z

**Published:** 2021-06-17

**Authors:** Armin R. W. Elbers, José L. Gonzales

**Affiliations:** Department of Epidemiology, Bioinformatics and Animal Studies, Wageningen Bioveterinary Research, P.O. Box 65, 8200 AB Lelystad, The Netherlands

**Keywords:** Diseases, Medical research

## Abstract

In the Netherlands, free-range layer farms as opposed to indoor layer farms, are at greater risk with regard to the introduction of avian influenza viruses (AIVs). Wild waterfowl are the natural reservoir hosts of AIVs, and play a major role in their transmission to poultry by contaminating free-range layer areas. The laser as a wild bird repellent has been in use since the 1970s, in particular around airfields to reduce bird-strike. The efficacy of laser for reducing wild bird numbers in and around free-range poultry areas has however not been investigated. During the autumn–winter, wild bird visits to the free-range area of a layer farm was surveilled by video-camera for a month without laser, followed by a month with laser. The automated laser (Class-III B qualification) was operated in two separate areas (i) within the poultry free-range area that directly bordered the poultry barn between 5:00 p.m. and 10:00 a.m. when poultry were absent (free-range study area, size 1.5 ha), and (ii) in surrounding grass pastures between 10:00 a.m. and 5:00 p.m. The overall (all bird species combined) efficacy of the laser for reducing the rate of wild birds visiting the free-range study area was 98.2%, and for the Orders Anseriformes and Passeriformes, respectively, was 99.7% and 96.1%. With the laser in operation, the overall exposure time of the free-range area to wild bird visits, but specifically to the Order Anseriformes, was massively reduced. It can be concluded that the Class-III B laser is highly proficient at keeping wild birds, in particular waterfowl, away from the free-range area of layer farms situated along a winter migration flyway.

## Introduction

Wild birds, in particular waterfowl, are the natural reservoir hosts of low pathogenic avian influenza viruses (LPAIVs), and are implicated in the long-distance dissemination of both LPAIVs and highly pathogenic avian influenza viruses (HPAIVs)^1,2^. Surveillance studies in birds of the orders Anseriformes (several species of swans, wild ducks and geese) and Charadriiformes (several species of waders, gulls and terns) indicate high LPAIV prevalence, especially in dabbling ducks^3–8^. For this reason, and because many migrate in spring to return in early winter, these are AIV high-risk orders^5,9,10^. Poultry farms with a free-range area and surrounded by grass pastures that are situated close to waterways and nature areas, are an interesting habitat for foraging wild waterfowl that can cause grazing damage. In the Netherlands, the risk of introduction of LPAIV and HPAIV is higher on free-range layer farms compared to layer farms where the poultry are housed permanently^11–15^. On free-range layer farms, poultry become infected outdoors either directly via contact with wild birds visiting the free-range area, or indirectly, by drinking from pools of rainwater contaminated by wild bird faeces^16,17^. Another indirect AIV transmission pathway involves poultry coming into contact with wild bird droppings that may litter free-range soils^18^.

Laser (**L**ight **A**mplification by **S**timulated **E**mission of **R**adiation) has since the 1970s been used around airfields as a repellent to reduce bird-strike^19–22^. Subsequently, lasers were deployed also against birds causing damage to agricultural crops^23–25^, and to disperse nuisance birds at landfills, on oil-rig platforms, and in recreational parks^26–30^. Earlier studies investigating the efficacy of laser in relationship to wild birds were sometimes executed in conditioned experiments using confined rooms or cages with a limited number of captured wild birds. The duration of these experiments was quite limited (few days)^21,23,24,26,28,31–33^. Most of these studies, using different quality of lasers, reported avoidance behavior of the birds in connection to the laser beam in more than 80% of the experiments, particularly for water birds (geese and gulls). Wild birds perceive the rapid movement of the laser beam across the ground as a physical threat and react by vacating the area. The eyes of nocturnally active birds such as the mallard (*Anas platyrhynchos*) are able to adjust to low-light conditions and react strongly to the light of a laser^34^.

Wild bird visits to the free-range area of a layer farm have been surveilled previously using a video-camera recording system (VCRS)^35^. Between January and August, several species of gulls visited almost daily from sunrise to 10:00 a.m. whereas, in contrast, mallards would predominate between December and February, visiting the free-range area from sundown till dawn. Video surveillance provided no evidence for direct contact between layers and wild birds in the free-range area. Indeed, nearly all wild birds were observed to leave the free-range area as soon as the layers began to appear from out of the poultry barn in the early morning. This behaviour indicated that the most likely route of AIV take-up in layers outdoors was via water or soil in the yard becoming contaminated by wild bird droppings.

Reducing visits of wild birds to the free-range area seems to be a logical measure preventing poultry from coming into contact with potentially contaminated soil and water. To our knowledge, on free-range layer farms, the use of laser to reduce wild bird visits has yet to be tested in a scientific setting. Hence, the objective of our study was to determine the efficacy of laser for reducing wild bird visits to a layer farm situated in an avian influenza hotspot of the Netherlands.

## Materials and methods

### Free-range layer study farm

**Figure 1:**
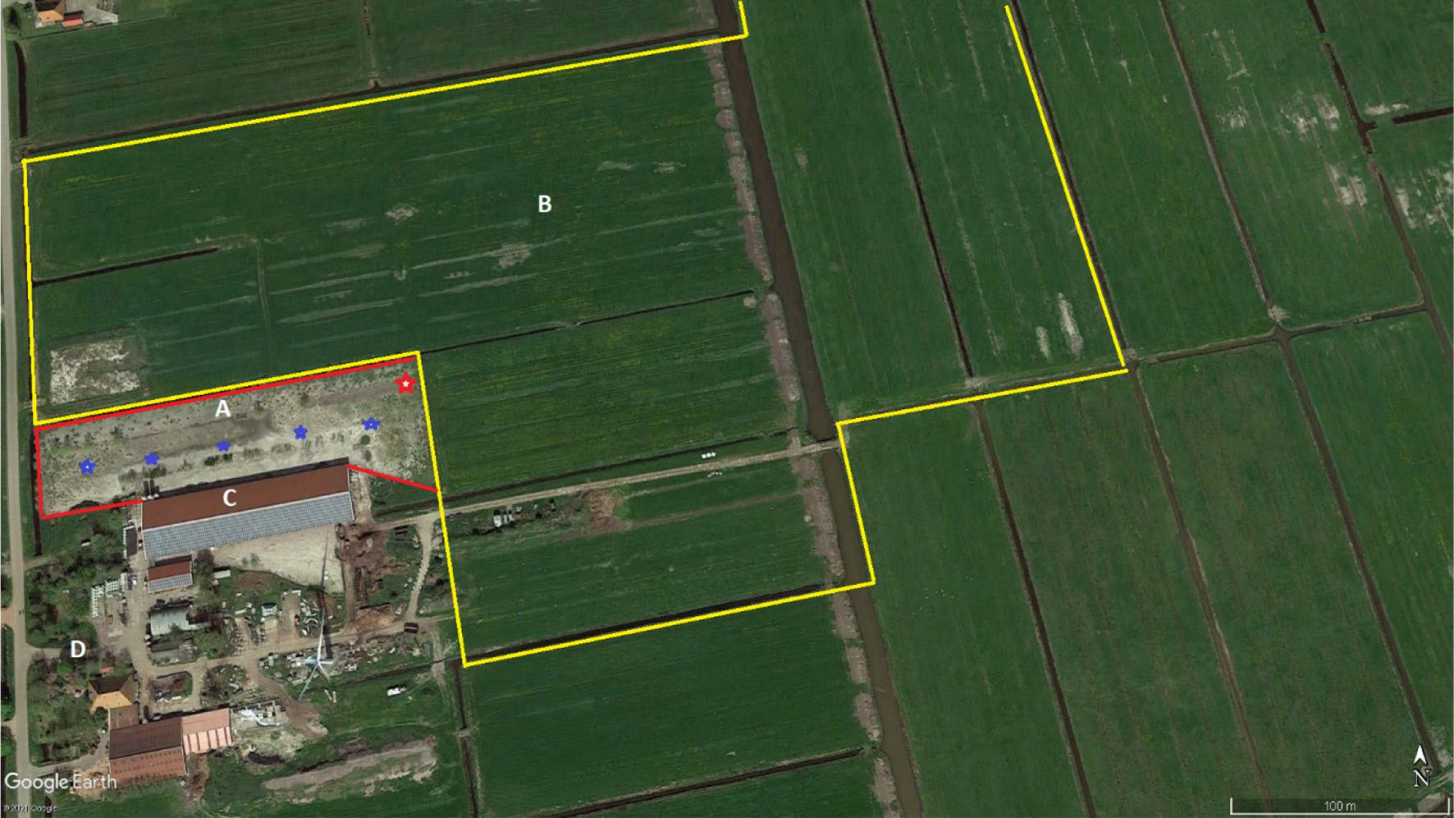
Aerial view of the layer farm, with the free-range study area (A) directly connected to the poultry house (C), and the surrounding grass pastures (B) that formed the total surface of the free-range area (A + B). The red line marks the fence. The area within the yellow line (B) is the range of the laser during daylight laser beaming. The area within the red line (A) is the range of the laser during night laser beaming. The red star (upper right corner in area A) indicates the position of the laser. The blue stars in area A indicate the position of the wide-angle video-cameras. The farm house is indicated by D (source: Google Earth, 2021 Google).

The free-range layer farm was selected in an earlier study^35^ to investigate visits of wild fauna to the free-range poultry yard using a VCRS. The study farm selected has experienced several LPAIV introductions in the 12 years since production started. As part of the national surveillance program, all free-range layer farms in the Netherlands, every three months, undergo serological tests for the presence of AIV antibodies^15^. On the study farm, about 38,000 layer hens are involved in egg production, and are housed nightly in a barn measuring approximately 120 m × 40 m (Fig. 1). According to regulations^36^, within the free-range area outdoors, each chicken must have access to at least 4 m^2^ of free space. The part of the free-range area directly connected to the poultry barn is approximately 1.5 ha in size and is partly fenced (Fig. 1); we refer in the rest of the manuscript to this area as the free-range study area in which the VCRS was installed. In spite of the existence of an opening that connects the free-range study area and the surrounding grass pastures, no layers were seen to ever go there. This is not unusual: in general, only a small proportion of the layer flock uses the free-range area at any given moment. In addition, most of the poultry in the free-range area, though patchily distributed, invariably stay close to the barn^37–39^. Layers access the free-range study area through pop holes in the barn’s side-wall.

In the free-range study area 22 willow trees provide the chickens with cover, shade and shelter, both from the weather and from predators. The farmer’s home is surrounded by some large trees and situated close to the barn (Fig. 1). In the yard, several 1 m wide water ditches exist and are connected to larger waterways outside (Fig. 1). The layers use the free-range study area daily between 10:00 a.m. and sundown. Layers are never fed and watered outdoors. Fifty sheep, and a cattle herd comprising 60 dairy cows and 60 young stock, occur elsewhere on the farm.

### Video-camera recording system (VCRS)

**Figure 2:**
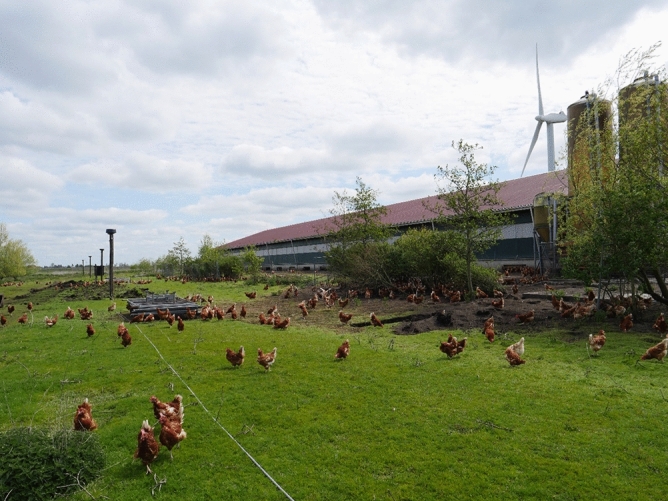
Wide-angle video-cameras installed at a height of approximately 4 m above ground-level on poles in the free-range study area (source: photo by first author).

The free-range study area was surveilled using eight Hikvision low-light Turret 4Mp video-cameras, each fitted with a fixed focus wide-angle 2.8 mm lens (Hikvision, Hangzhou, China; www.hikvision.com). The cameras were positioned on poles at a height of 4 m above ground-level (Fig. 2). It was not possible to position cameras in the grass pastures that lay adjacent to the free-range study area, and which extended hundreds of meters away from the barn. Thus, the impact of laser on wild birds in the grass pastures is assessed anecdotally, i.e., is based solely on observations made daily by the farmer.

The VCRs were connected to a TruVision NVR10 network video recorder with HDMI/VGA video-output and a 12 Tb hard disk for storage (Interlogix, United Technologies Corporated, NC, USA; www.interlogix.com/truvision). The recording of wild bird activity in the free-range study area was done at 2 frames/s and under low-light conditions was facilitated using IR LEDs.

### Laser equipment and applications

**Figure 3:**
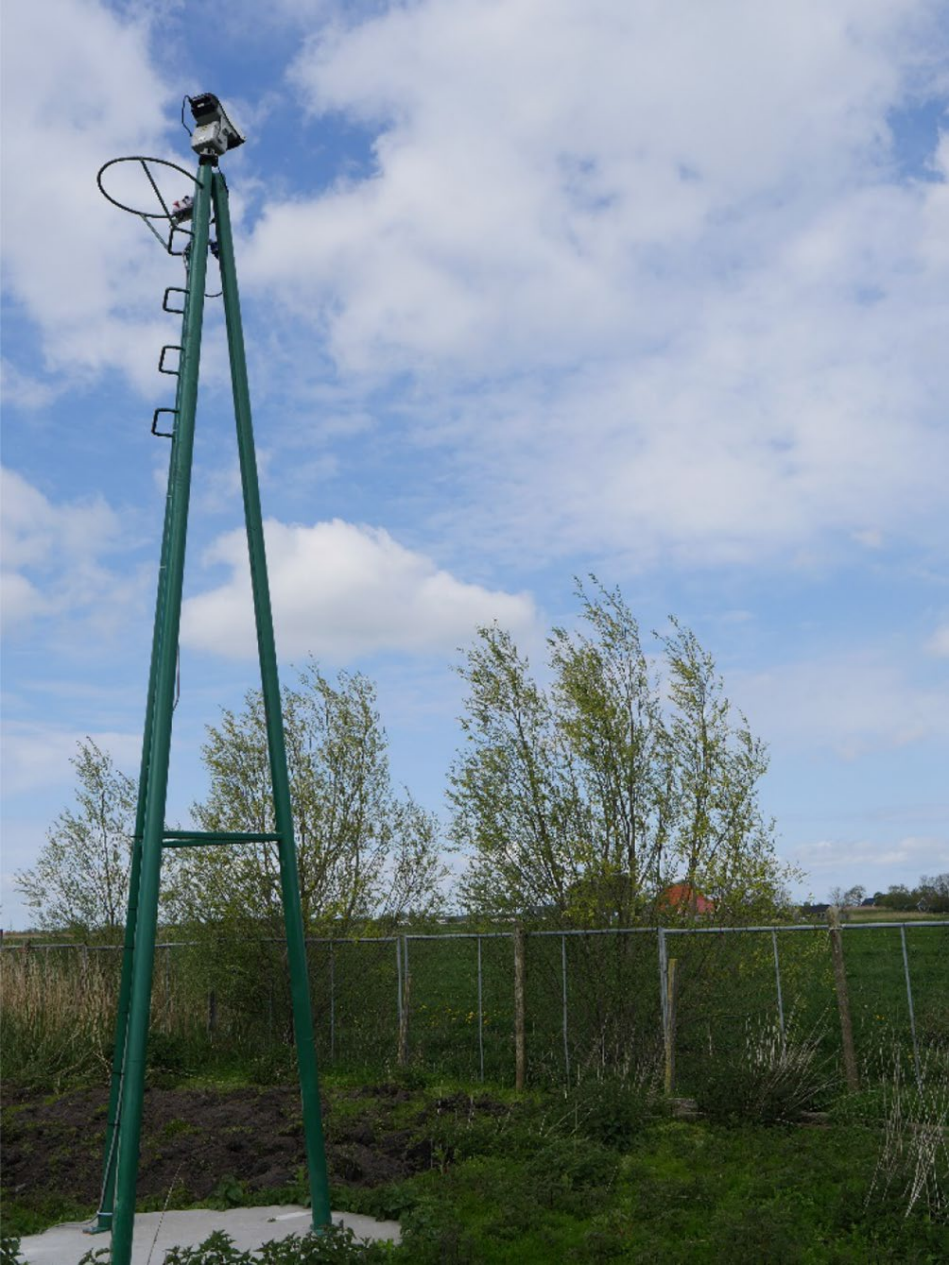
The laser placed on a steel, three-legged tower (the top was 6 m above ground level) that was secured to a concrete slab (1.5 m × 1.5 m) weighing approximately 2000 kg (source: photo by first author).

An automated laser (Laser Class-III B), Avix Autonomic Mark II (www.birdcontrolgroup.com, Delft, The Netherlands) was used. The laser was affixed to the top of a steel, three-legged tower 6 m in height. The tower was secured to a concrete slab (1.5 m × 1.5 m) weighing approximately 2000 kg (Fig. 3) and positioned in the right upper corner of the free-range study area (Fig. 1). The laser wavelength is 532 nm (green), and has a variable power (50–499 mW). The avian retina functions within a spectral sensitivity range of approximately 300–700 nm^40^. In general, the A Class-III B laser is only considered hazardous when the beam is stared into directly and at distances close to the diffuser^41^. By law, the use of the laser must remain confined to the owner’s landholding, and the beam is not allowed to penetrate onto public pathways and roads, and into neighboring buildings. A signpost warning the public is required: “Class-III B laser controlled area—avoid eye or skin exposure to direct or scattered radiation”. Safety requirements are laid down in European Directives 2001/95/EC, 2006/25/EC and 2014/59/EC and for instance for the United States in the American National Standards Institute (ANSI) Z136 series of standards.

The laser has a horizontal projection range of 360° (continuous), and a vertical projection range of − 50° to + 30°. The beam projects a distance of 100 m for every meter the laser is positioned above ground level. Beam movement is computer-guided and is programmed by manual-visual selection of geographical waypoints; this allows the beam to move smoothly and automatically between one programmed waypoint and the next.

The free-range study area was split into two parts and laser-beamed separately: one part in 3:30 min, the other part in 5:30 min, interrupted by a laser-free period of 1 min. The area was surveilled daily from 5:00 p.m. in the evening—depending on the change in daylength over time—until 10:00 a.m. the following morning, the time when poultry would begin filter out of the barn into the free-range study area. The grass pastures—up to a distance of 600 m (see Fig. 1)—were laser-beamed between 10:00 a.m. in the morning and approximately 5:00 p.m. in the evening. The pastures were laser-beamed in 10 parts, leaving an interval of 1 min between each part; each ten-part cycle lasted approximately 45 min. Only the free-range study area was laser-surveilled at night.

### Presence of wild birds in surroundings

The study farm is situated in the north of the country near to the coast in an area famed for its flocks of migrating waterfowl that annually fly either up or down the coast, in spring, autumn, and winter. Accordingly, particularly during autumn and winter, flocks of multiple wild bird species appear in the waterways and grass pastures that surround the free-range study area. These flocks include the barnacle goose (*Branta leucopsis*), the greater white-fronted goose (*Anser albifrons*), the greylag goose (*Anser anser*), the mute swan (*Cygnus olor*), the Eurasian wigeon (*Mareca penelope*), the mallard (*Anas platyrhynchos*), the black-headed gull (*Chroicocephalus ridibundus*), the lesser black-backed gull (*Larus fuscus*), the cormorant (*Phalacrocorax carbo*) and the oyster catcher (*Haematopus ostralegus*)^35^.

### Translating video recordings into data for analysis

The method used to video-record and store information around wild bird movements was described previously^35^. The information databased included the following: observation date; laser in operation in the free-range yard or not; wild bird identity (Family, Order, Species); time (hh:mm:ss); wild bird(s) start presence in the free-range study area; time (hh:mm:ss) wild bird(s) departure from the free-range study area; total number of wild birds visiting. A unique wild bird visit was defined as that in which one or more individuals of a wild bird species either landed in, or sat on the fence, and recording the length of time it remained in situ before departing^35^. Wild birds visiting per observation day by species was defined as the total number of wild birds in visits per observation day by species. The daily period of exposure of the free-range study area to wild birds, was calculated for each species as the number of individuals counted multiplied by total visiting time (of the species in question).

### Study design

According to the manufacturer, the laser is able to decrease wild bird visits by 75% or more. Based on observations made previously^35^, on average 8 wild ducks [standard deviation (s.d.): 10 ducks] visited the free-range study area in the period November–February (in the absence of laser). With 95% confidence, and 80% statistical power, taking the above-mentioned expectations into account, the calculated minimum sample size (number of observation days) needed to test the efficacy of the laser to keep wild birds—in particular ducks—out of the free-range study area, is 24 days with (treatment period) and 24 days without the use of a laser (control period)^42^. The study design was as follows: commence with approximately one month of video-camera surveillance of wild bird visits without laser (December 2019: sunset varying between 4:28 p.m. and 4:35 p.m.; sunrise between 8:21 a.m. and 8:48 a.m.), followed by one month of observations using laser (January 2020: sunset varying between 4:35 p.m. and 5:25 p.m.; sunrise between 7:52 a.m. and 8:48 a.m.). We chose December and January because previously^35^ these were the months during which daily visits in birds of the Order Anseriformes peaked. In addition, there was no difference in the average/median number of birds visiting monthly, meaning the influence of natural temporal changes on bird numbers visiting is negligible.

**Figure 4:**
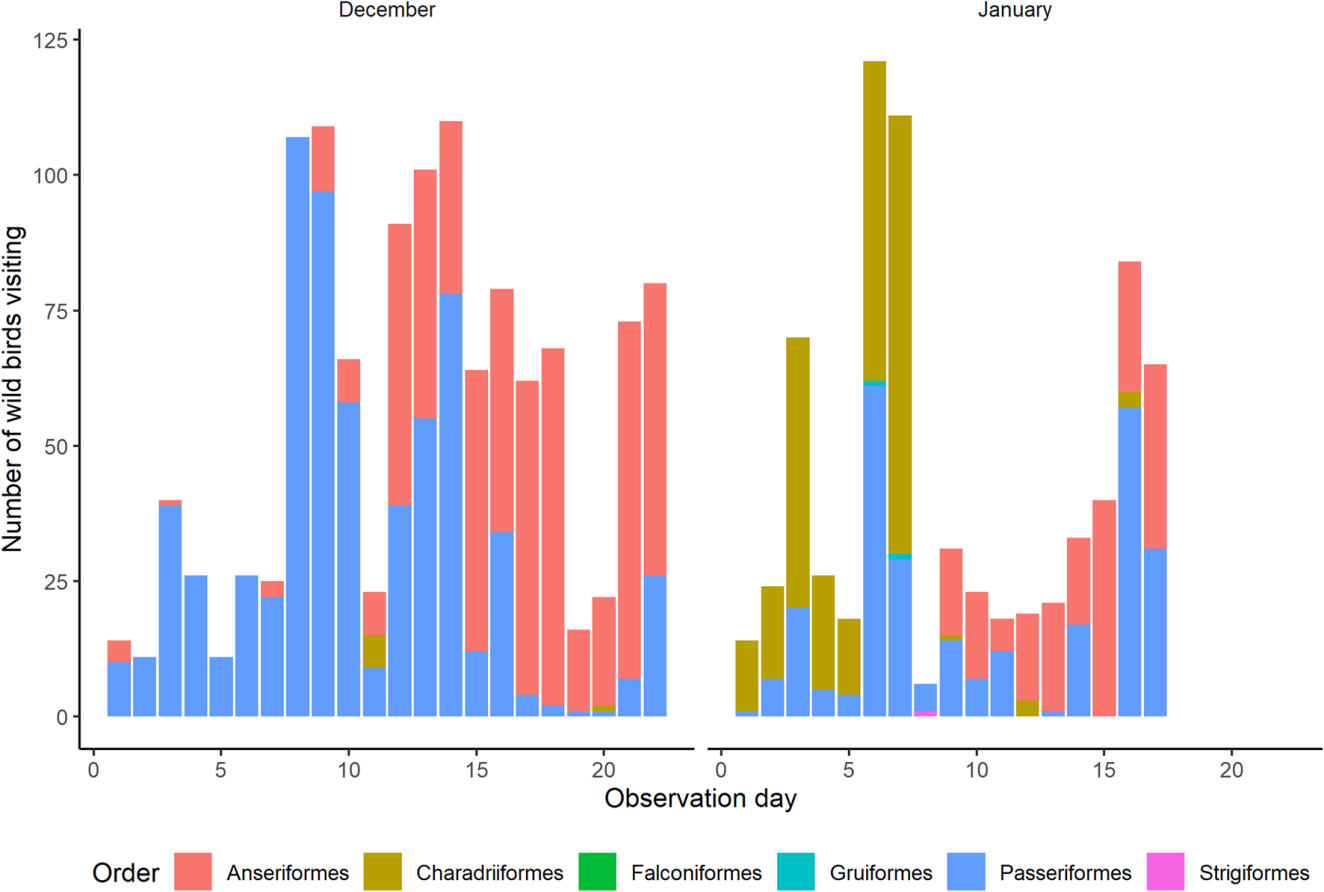
Number of wild birds visiting the free-range study area by observation day and wild bird Order in the months December and January during a previous study at the same study farm (Elbers & Gonzales, 2019).

Additionally, a sensitivity analysis was performed: laser efficacy was assessed by comparing wild bird visitation rates with the laser in operation in this study to visitation rates in periods without a laser in operation (December and January months) in our previous study^35^ (Fig. 4).

### Observer agreement

Two observers were involved in converting the video recordings into analyzable data. The Field Guide to the Birds of Europe^43^ was used in consultation with an experienced ornithologist where necessary. In those instances where a species could not be identified with certainty, the particular observation was designated ‘unspecified’. Inter-observer agreement as a measure of data quality was calculated using Cohen’s Kappa statistic^44^, in which the two observers independently note wild bird visits for the same observation days. Cohen’s Kappa was calculated separately for (i) the night of visits by AI risk birds, namely ducks, and (ii) for other avian visitors, such as song birds, which may appear between sunrise and 10:00 a.m., i.e., in the early hours preceding the appearance of the chickens in the free-range study area. Based on an a priori estimated inter-observer agreement of approximately 0.95 with a maximum acceptable error of 0.10, and a 95% confidence level, a sample size of approximately 16 observation days is required^42^.

### Statistical analysis

To assess laser efficacy, the wild bird visitation rate per observation day was compared between two periods: ‘with laser’ (treatment) versus ‘without laser’ (control). To estimate these rates and to compare them, a negative binomial regression model was fitted, which corrects for overdispersion observed when fitting a Poisson regression model, using the library MASS^45^ in the statistical software package R version 4.0.2^46^. Two models were fitted, the first model aimed to compare the overall wild bird visitation rate between periods with and without the laser in operation. In this model the response variable was the total numbers of birds visiting per observation day and the explanatory variable was a variable identifying the presence or absence of the laser. The exponent of the estimated regression coefficient of this model is the relative risk (RR) of birds visiting the free-range study area when the laser was present.

The second model fitted used as explanatory variables the taxonomic Order of the birds visiting and the interaction with the variable identifying the presence/absence of the laser. The exponent of the bird order-laser presence interaction represents the RR of birds from each Order visiting the free-range study area when the laser was present. The threshold for significance of the estimated parameters was p < 0.05. Next we estimated the percentage efficacy of the laser for reducing the daily rate of wild birds visiting as: Laser efficacy = (1 − RR_Laser) × 100.

Akaike’s Information Criteria (AIC) were used as a model selection criterion. Model fit was assessed by variation explained by the model (R^2^) and by residual analysis.

Figures with descriptive data showing wild bird visitation rates with and without use of the laser were designed using the library ggplot2^47^ in the statistical software package R.

Comparisons between the period with the laser absent (this study) and present were also made on the number of days with observed wild bird visits within each experimental period and the free-range exposure time. Number of days with visits were compared using Fisher’s exact test. Exposure time was compared by fitting negative binomial regression models using the same approach as that described for assessing the rates of wild birds visiting per day.

### Ethical statement

Based on the criteria of EU directive 2010/63/EU on animal experimentation and in accordance with the criteria of the institute’s Animal Welfare Body (AWB) of Wageningen Research, it was considered that this experiment is not falling under the EU directive. There was no handling introducing harm to animals or any direct human contact with wild fauna. The use of the laser was considered not to cause pain, suffering or lasting harm to wild birds; wild birds perceive the rapid movement of the laser beam across the ground as a physical threat and react by vacating the area before the laser beam would reach them. The reporting of this study in the manuscript follows the recommendations in the ARRIVE guidelines (https://arriveguidelines.org).

## Results

### Inter-observer agreement

Mean Cohen’s Kappa statistic measuring the inter-observer agreement for observations on mallards was 1.00 (s.d. 0; range 1–1) based on 17 observation days. For observations on other wild birds, mean Cohen’s Kappa was 0.994 (s.d. 0.027: range 0.89–1) based on 17 observation days with these birds present. Both measures of inter-observer agreement indicate perfect agreement^44,48^. Disagreement was present in only one wild bird observation on a given day where some White wagtails (*Motacilla alba*) were missed by one of the observers.

### General observations

Mallards (*Anas platyrhynchos*) visited the free-range study area without the laser in operation from shortly after sunset (when the layers were already back into the barn) to sunrise. The Western barn owl (*Tyto alba*) hunted in the free-range yard for mice and rats at night without the laser in operation. Visits to the free-range study area by the Blackbird (*Turdus merula*), Western yellow wagtail (*Motacilla flava*), White wagtail (*Motacilla alba*), Common Moor-hen (*Gallimula chloropus*) and Common kestrel (*Falco tinnunculus*) were limited to the period between sunrise and 10:00 a.m. when the laser was not operational. These wild birds took flight when the layers began to appear in the free-range study area at around 10:00 a.m. Though gulls of different species were expected to appear in the free-range area between sunrise and 10:00 a.m., this was never the case; the gulls were instead found in the surrounding pastures when the laser was only operational in the free-range study area.

**Figure 5:**
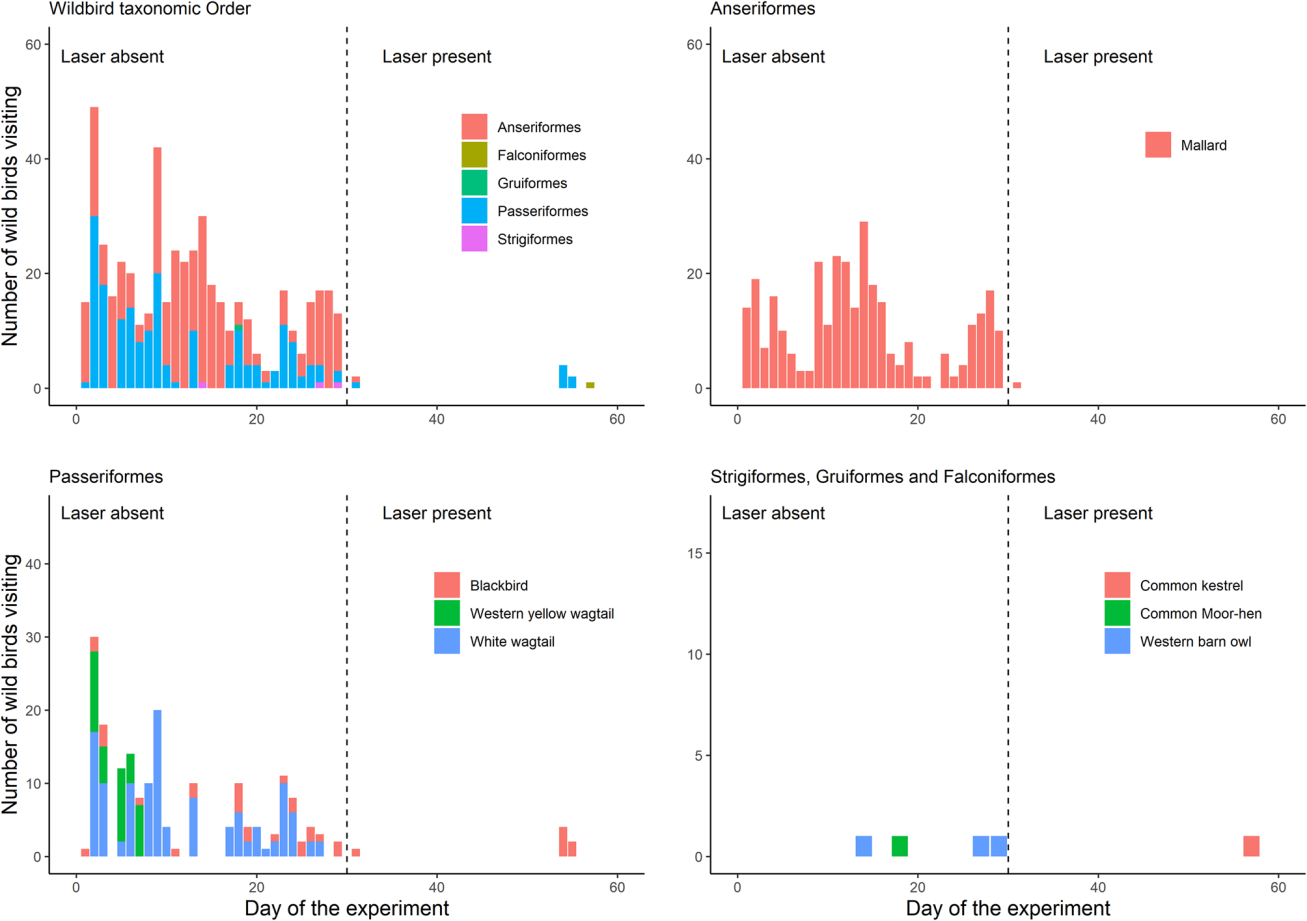
Number of wild birds visiting the free-range study area of the layer farm by observation day with and without the laser in operation; for all wild bird species together (upper left corner); for wild birds of the order Anseriformes (upper right corner); for wild birds of the order Passeriformes (lower left corner); for wild birds of the order Strigiformes, Gruiformes and Falconiformes grouped together (lower right corner).

Without the laser in operation, mallards visited the free-range study area on 28 of the 29 observation days, whereas with the laser in operation, they visited on only 1 of 28 observation days (Fig. 5). This reduction is highly (Fishers’s exact test, p < 0.001) significant. Without the laser in operation, one or other of the following bird species, namely the blackbird, the Western yellow wagtail, the White wagtail, the Western barn owl, the common moorhen, and the Common kestrel, were observed to visit the free-range study area on 18 out of 29 observation days, whereas with the laser in operation, they visited it on only 4 of 28 observation days. This represents a highly (Fisher’s exact test, p < 0.001) significant reduction in the number of days wild birds visited the free-range study area.

A stone marten (*Martes foina*) family—with a burrow in the middle of the free-range study area—and numerous unspecified species of mice and rats appeared daily during the night in the free-range study area. The laser had no influence whatsoever on the daily rate of visiting by these mammals.

### Daily wild bird visitation rate

A summary of descriptive statistics on daily wild bird visitation rates with and without the laser in operation, and grouped by taxonomic Order, is provided in Table 1. Wild birds of the Order Anseriformes, in this case only mallards, visited the free-range study area often and with considerable day-to-day variation in the period without the laser in operation while virtually no mallards were observed when the laser was in operation (Fig. 5). A similar effect of the laser can be observed for visiting wild birds of the Orders Passeriformes, Strigiformes, Gruiformes and Falconiformes, although the numbers of their visits was considerably less compared to that made by mallards (Fig. 5). The estimated mean daily wild bird visitation rate (all species) was 3.51 [95% Confidence Intervals (CI) 2.35–5.53] when the laser was absent. This rate was 55 times (1/0.0183; p < 0.001) lower when the laser was used: RR = 0.0183 (95% CI 0.007—0.043). A similar significant reduction (p < 0.001) in the mean daily wild bird visitation rate (all species) was observed when using the months of December and January from the previous study as control periods (Table 2).

**Table 1:** Summary of daily numbers of wild birds visiting during the experimental periods (28 observation days with and 29 days without the laser in operation), grouped by taxonomic Order, observed in the free-range study area.

Order	Laser present	Median	Min	Max	Estimated rate (95% CI)^a^
Anseriformes	No	10	0	29	10.93 (7.75–15.96)
	Yes	0	0	1	0.04 (0.01–0.26)
Passeriformes	No	4	0	30	6.34 (4.37–9.20)
	Yes	0	0	4	0.25 (0.11–0.27)
Falconiformes	No	0	0	0	0.05 (0.02–0.13)^b^
	Yes	0	0	1	0.01 (0.00–0.08)^b^
Gruiformes	No	0	0	1	
	Yes	0	0	0	
Strigiformes	No	0	0	1	
	Yes	0	0	0	

^a^Estimated mean rates of visits [95% Confidence Intervals (CI)] using a generalized linear model with negative binomial distribution.

^b^These rates represent the estimated rates of visits for the three Orders Falconiformes, Gruiformes, Strigiformes combined.

**Table 2:** Overall relative risk ratios (RR) and efficacy of the laser to reduce wild birds visits.

Control	RR	95% CI	Efficacy %	95% CI	R^2^ Nagelkerke^a^
December (this study)	0.018	0.007–0.043	98.17	95.69–99.28	0.48
December	0.007	0.002–0.020	99.31	98.02–99.77	0.48
January	0.009	0.003–0.024	99.09	97.56–99.69	0.54

^a^These measures indicate the overall performance of the generalized linear models; (negative binomial distributions) used to quantify the RR and Efficacy.

### Daily wild bird visitation rate by taxonomic order

Mean daily visitation rate of wild birds of the Order Anseriformes was 306 times (1/0.00327; p < 0.001) lower when the laser was used: RR = 0.00327 (95% CI 0.00018–0.0159), whereas that of the Order Passeriformes was 25 times (1/0.0394; p < 0.001) lower when the laser was used: RR = 0.0394 (95% CI 0.0149–0.0919) (Table 3). Due to the low number of daily visits, the RR for birds of the Orders Falconiformes, Gruiformes and Strigiformes combined was approximately only about 4 times lower when the laser was operational, i.e., not significant (p = 0.22) (RR = 0.256; 95% CI 0.0129–1.7689) (Table 3).

**Table 3:** Relative risk ratios (RR) and efficacy of the laser to reduce wild birds visits estimated by taxonomic Order.

Order	Control	RR	95% CI	Efficacy %	95% CI	R^2^ Nagelkerke^a^
Anseriformes	December (this study)	0.003	0.000–0.016	99.67	98.4–99.98	0.96
	December	0.001	0–0.008	99.86	99.19–99.99	0.96
	January	0.003	0–0.034	99.9	98.9–100	0.63
Passeriformes	December (this study)	0.039	0.015–0.091	96.06	90.81–98.51	0.96
	December	0.008	0.003–0.023	99.19	97.69–99.74	0.96
	January	0.016	0.002–0.099	98.43	90.08–99.81	0.63
Other	December (this study)^b^	0.256	0.013–1.770	74.4	0.00–98.70	0.96
	December^c^	0.150	0.008–0.891	85.03	10.93–99.22	0.96
	January^c^	0.003	0–0.018	99.69	98.16–99.98	0.63

^a^These measures indicate the overall performance of the generalized linear models (negative binomial distributions) used to quantify the RR and Efficacy; the three R^2^ values are given for each of three models (Number of birds ~ wild bird Order + Laser_present:Order) fitted for each of the scenarios: (1) this study, (2) December previous study as control, (3) January previous study as control.

^b^The Orders Falconiformes, Gruiformes and Strigiformes were grouped as “Others”.

^c^The Orders Charadriiformes, Falconiformes, Gruiformes and Strigiformes were grouped as “Others”.

The RR risk estimates using data from our previous study were similar, particularly when estimations were done using the month of December as control (Table 3). Similarly, only the RR for the Orders grouped as “Other” was not significant (p = 0.08). When estimates were done using the data from January, the RR for the Orders “Other” became significant (p < 0.001), which could be attributed to the large number of birds of the Order Charadriiformes visiting the free-range study area in January that year. In the current study, wild birds from the Order Charadriiformes were only observed in January in the morning (between sunrise and 10:00 a.m.) in the pastures next to the free-range study area, which were not laser-beamed at that time of the day.

### Laser efficacy

The estimated RRs were used to calculate the laser’s percent efficacy for reducing wild bird visitation rates. For all species combined it was 98.2% (95% CI 95.7–99.3), with similar estimates obtained when estimating efficacy using data from our previous study (Table 2). Laser efficacy estimates per Order were as follows: Anseriformes 99.7% (95% CI 98.4–99.98), Passeriformes 96.1% (95% CI 90.8–98.5), and Falconiformes, Gruiformes and Strigiformes combined 74.4% (95% CI 0–98.7) (Table 3).

Estimated laser efficacies were similar for birds of the Order Anseriformes and Passeriformes, regardless of the period (this study or previous study) used as control. However for the Orders grouped as “Other”, as a result of the observed differences in RR mentioned before when using the data from January of the previous study, laser efficacy as high as the efficacies estimated for birds of the Order Anseriformes and Passeriformes was observed (Table 3).

### Exposure time of free-range study area to wild birds

A summary of the descriptive statistics on exposure time of the free-range study area to visiting wild birds with and without the laser in operational, and grouped by taxonomic Order, are provided in Table 4. When the laser was operational, the overall exposure time (all bird species combined) was 617 times (1/0.00162; p < 0.001) lower (RR = 0.00162; 95% CI 0.000295–0.0089); for the Order Anseriformes it was 2320 times (1/0.000431, p < 0.001) lower (RR = 0.000431; 95% CI 0.0000246–0.00756); for the Order Passeriformes 17 times (1/0.0575; p < 0.001) lower (RR = 0.0575; 95% CI 0.0129–0.2566); and for the Orders Falconiformes, Gruiformes and Strigiformes combined, 18 times (1/0.0544; not significant, p = 0.14) lower (RR = 0.0544; 95% CI 0.00115–1.04175).

**Table 4:** Summary of daily exposure time (in min.) of free-range study area to wild birds. This is a combination of the number of birds visiting daily and the duration of their visit during the experimental periods (28 observation days with and 29 days without the laser in operation), grouped by taxonomic Order.

Order	Laser present	Median time	Min	Max
Anseriformes	No	2144.21	15.80	15,323.45
	Yes	1.25	1.25	1.25
Passeriformes	No	31.75	0.37	253.43
	Yes	4.43	1.00	5.05
Falconiformes	Yes	0.45	0.45	0.45
Gruiformes	No	1.88	1.88	1.88
Strigiformes	No	2.68	0.70	27.83

## Discussion

The overall (all bird species) efficacy of the laser for reducing the wild bird visitation rate was 98.2%. For birds of the Order Anseriformes, Passeriformes and the Orders Falconiformes, Gruiformes, Strigiformes and Charadriiformes grouped together, the laser efficacy was 99.7%, 96.1% and 74.4%, respectively. The overall exposure time of the free-range study area to all species of wild birds, but specifically to birds of the Order Anseriformes, was massively reduced. Taking into account the observations made previously by us on the very same farm^35^, there is little doubt that the marked reductions in wild bird visitation rates overwhelmingly can be attributed to the effectiveness of the laser. This leads us to conclude that the Class-III B laser is highly proficient for keeping wild birds—in particular wild ducks—away from poultry free-range areas.

As noted, the grass pastures surrounding the poultry free-range area were not monitored by VCRS. Instead, the farmer made a daily record of any wild bird activity he was to observe. When the laser was not operational, geese and gulls were observed to aggregate in large numbers on the pastures, but would quickly vacate them once the laser was made operational. Grazing damage by wild ducks and geese was completely stopped.

This field experiment was performed in a farm which has suffered multiple introductions of avian influenza virus along the years. The specific months of December and January were selected because these are not only the months with the highest risk for AIV-introduction in free-range layer farms in the Netherlands^49^, but also the months with the expected highest number of visitations to the free-range area by wild birds, especially mallards^35^. By selecting this farm, for which we had in-depth knowledge on its temporal exposure to wild birds, and using a before (control—no laser) and after design (intervention—with laser), we controlled for geographical and farm management sources of variation (e.g. different wild bird species and densities). This variation could have been introduced by using a different farm, in a different location, as control. In addition, availability of detailed data from a recent study allowed us to also assess efficacy of the laser by using as controls these previous observations. Our study design contributed to an increase of the statistical power of the study and reduction of the risk of bias, increasing therefore our certainty in the efficacy estimates.

Whilst our study design has its strengths, the use of only one farm limits the geographical and seasonal generalizability of the study. Our experiment comprises only one farm and the efficacy estimates may be representative for the wild bird species that visited or are present around this study farm. This may explain the differences in efficacy for the group “Other” in the sensitivity analysis (Table 3). Geographical variation in efficacy may be expected as a function of the wild bird species populations present in other regions. Other studies assessed laser efficacy on different bird species. These studies, although of different study design and use of other laser equipment, reported high efficacies (> 80%) specifically for birds of the Order Anseriformes^26,28,32,33^, Charadriiformes^21^, Passeriformes and Suliformes^23^. As for seasonality, this may also be linked to the seasonal distribution of wild bird species and densities in different regions and duration of day light. Our efficacy estimates may be limited to the Dutch winter period, which is the period of higher risk for AIV-introduction in poultry^48^. In summary, our study together with the above mentioned studies indicate an expected high efficacy of lasers to keep away wild birds in the winter period, particularly birds of the Order Anseriformes and Charadriiformes.

Wild birds may become habituated to the presence and operation of the laser, in particular when the stimulus becomes repetitive and predictable^50,51^. Programming the beam of the laser to move arbitrarily across the landscape, has the effect of making it unpredictable and therefore less likely to be ‘accepted’ by wild bird. Once habituated, wild birds will: (i) continue to avoid the area during operation of the laser, directly affecting its usefulness, and (ii) when the lasering ends, the amount of time it takes for wild birds to return may increasingly shorten. For these reasons, a study on habituation conducted over the longer term (for several months and over consecutive seasons) is needed, but will be difficult to execute and at considerable cost^24^. Werner and Clark^32^ conducted 5-day and 20-day laser trials against Canada geese (*Branta canadensis*). During the experiments, the geese simply avoided the experimental area, returning soon after lasering ended. Under controlled conditions, Blackwell et al.^26^ lasered caged groups of 4–6 mallards and Canada geese for several consecutive periods, each lasting 20–80 min. All the birds exhibited clear avoidance behaviour; remarkably, the mallards began to show signs of habituation. In the field, Latour and Stahl^24^ used a handheld laser against Greylag geese; though the birds did not exhibit habituation, they too were observed to return to the area less than a day after lasering had ended. Glahn et al.^31^ conducted five field trials using laser in an attempt to deter cormorants from roosting in trees; each trial lasted three nights. The laser effectively reduced cormorant roosting by more than 90%, and no signs of habituation were observed. Compared to these investigations, our study encompassed the longest use of the laser (28 days consecutively); in spite of its lengthy deployment, the wild birds were not observed to become habituated to the laser’s presence and operation. In addition, as observed by others in previous investigations^21,24,28,33^, wild birds dispersed by laser, were found to settle down in pastures nearby, and did so at a distance beyond that of the laser’s range. It is concluded, albeit indirectly, that the laser does not represent a wild bird hazard; this is predicated on the fact that wild birds would return to the area laser-beamed and do so within one or more days^21,23,26,32^. In spite of the fact that lasering was conducted continuously for a much longer period (28 days), the result remained the same: after lasering ceased, wild birds of the Order Passeriformes and Anseriformes, after two and six days respectively, again began to show up in the free-range study area, and in numbers comparable to those encountered prior lasering.

The impact of laser use on wild bird health is little studied. Using a hand-held 5 mW Class-III-B laser, Glahn et al.^31^ aimed the beam from various distances (1, 13 and 33 m) at the eyes of five captive double-crested cormorants (*Phalacrocorax auritus*). The exposure distances chosen were based on the nominal ocular risk distance (NORD) to humans for this specific laser (approximately 13 m) and assuming an eye-blink reaction time of 0.25 s. The cormorant eyes were examined 24- and 48-h post-treatment; no ocular damage was apparent. According to the manufacturer the risk of the laser causing injury to the eyes of birds is minimal because they react to the approach of the beam along the ground by flying away immediately.

Approximately 20% of the free-range layer farms of the Netherlands have faced one or more LPAIV-introductions in the years between 2007 and 2018^13,15^, with the peak of introductions observed in the period between November and February^49^. Layer farms that are situated in close proximity to waterways and nature reserves are at greatest AIV-introduction risk because waterfowl are attracted to both environments^15^. So, in the case of free-range layer farms situated in AIV high-risk areas and with a history of recurrent LPAIV-introductions, the laser might serve as a helpful preventive towards keeping waterfowl away from the free-range area of poultry layer farms.

The ecological consequences that may arise from the mass application of lasers in the poultry industry must first be investigated before any recommendations can be made and implemented. In the interim, the results of this study demonstrate the efficacy of the laser for reducing wild bird visits, and means that at present it could be put to preventative use, in particular on those AIV hot-spot poultry farms that since 2014 repeatedly have been troubled by the unforeseen introduction of HPAIV^52^. It should be noted that the laser will contribute to reduction of the risk of AIV-introduction via (in)direct contact with wild birds. Poultry farms may still experience other routes of AIV-introduction: between-farm transmission; rats and mice entering barns with AIV-contamination stuck to their fur or feet; unintended transport of AIV-contamination into the barn, originating from wild bird droppings stuck to e.g. farmers’ boots, etc. Therefore, maintenance of high levels of biosecurity is paramount.
